# Impaired L-type Ca^2+^ channel function in the dystrophic heart

**DOI:** 10.1186/2050-6511-13-S1-A41

**Published:** 2012-09-17

**Authors:** Xaver Koenig, Xuan B Dang, Lena Rubi, Ágnes K Mike, Péter Lukács, René Cervenka, Vaibhavkumar S Gawali, Hannes Todt, Reginald E Bittner, Karlheinz Hilber

**Affiliations:** 1 Department of Neurophysiology and Neuropharmacology, Center for Physiology and Pharmacology, Medical University of Vienna, 1090 Vienna, Austria; 2 Neuromuscular Research Department, Center for Anatomy and Cell Biology, Medical University of Vienna, 1090 Vienna, Austria

## Background

Duchenne muscular dystrophy (DMD), caused by mutations in the dystrophin gene, is an inherited disease characterized by progressive muscle weakness and degeneration. Besides the relatively well described skeletal muscle degenerative processes, DMD is associated with cardiovascular complications including cardiomyopathy and cardiac arrhythmias. The current understanding of the patho-mechanisms is still very limited, but recent research suggests, that dysfunctional ion channels in dystrophic cardiomyocytes considerably contribute to the cardiovascular complications.

## Methods

By using the whole cell patch clamp technique, the functional properties of voltage-gated L-type Ca^2+^ channels were studied in ventricular cardiomyocytes derived from normal and dystrophic mice. Physiological consequences were followed up by investigating action potentials and by comparing surface ECG recordings in wild-type and dystrophic mice. Besides the commonly used dystrophin-deficient mdx mouse model, this study is amongst the first to additionally include the dystrophin-utrophin double-deficient mouse model for DMD.

## Results

We found that the voltage-dependent inactivation of L-type Ca^2+^ channels is significantly reduced in dystrophic cardiomyocytes. Moreover, in cardiomyocytes derived from dystrophic adult animals, current density levels are significantly increased. Action potential duration was not prolonged in dystrophic murine cardiomyocytes, but incorporating the observed reduction in current density into a computer model of a human cardiomyocyte resulted in a marked prolongation. Physiological relevance was further suggested by an acceleration of atrioventricular nodal conduction and a prolongation of ventricular repolarisation in the ECG.

## Conclusions

L-type Ca^2+^ channels are significantly impaired in dystrophic cardiomyocytes and likely contribute to the cardiovascular complications associated with Duchenne muscular dystrophy.

## Background

This work was supported by the Austrian Science Fund (FWF, grant P23060).

